# Antifungal and enzymatic activities of endophytic fungi associated with *Tamarix nilotica*

**DOI:** 10.1080/15592324.2024.2439250

**Published:** 2024-12-29

**Authors:** Helal F. Al-Harthi

**Affiliations:** Biology Department, Turabah University College, Taif University, Turabah, Saudi Arabia

**Keywords:** Antifungal, *Alternaria alternata*, hydrolytic enzymes, ITS rRNA, *Penicillium chrysogenum*, wild plants

## Abstract

Fungal endophytes were recovered from *Tamarix nilotica* (Tamaricaceae) roots and stems collected in Taif, Saudi Arabia. A total of 49 different taxa were identified. The overall colonization rate of root and stem segments was 30.6%. A total of 49 isolates were collected and categorized into 21 operational taxonomic units using the rRNA gene’s internal transcribed spacer region. The most prevalent species were *Penicillium chrysogenum* (16 isolates), Fungal sp. (12), and *Alternaria alternata* (10). Forty-nine isolates were investigated for antifungal activity against *Fusarium solani* and *Rhizoctonia solani*; all tested isolates showed antifungal activity against *Fusarium solani*, while 43 isolates showed antifungal activity against *Rhizoctonia solani*. The most potent antifungal agents against *Fusarium solani* and *Rhizoctonia solani* are *Aspergillus ochraceus* (2 isolates) and *Penicillium chrysogenum* (16 isolates). Endophytic isolates collected during this experiment were evaluated to produce amylase, cellulase, lipase, and protease enzymes. Among the strains examined for enzymatic potentials (4 enzymes), 26 strains (53% of the total strains) produce only one enzyme. *Penicillium chrysogenum* (TUPc2, 3, and 4) produced the highest amount of the four enzymes that were examined.

## Introduction

Endophytic fungi are diverse and widely distributed in a variety of plants.^1–3^ Endophytic fungi and their host plants have a mutually beneficial symbiotic relationship because of their prolonged presence in plant tissues and coevolution. Additionally, certain endophytic fungi can increase resilience to abiotic stress, prevent diseases and pests, and stimulate host growth.^4–8^

The biotechnological community is interested in endophytic fungi because of their potential as a biological control agent^9–11^ and as a source of secondary metabolites beneficial for innovative drug development.^12^ Several studies^13–22^ have examined the antifungal and antibacterial activities of plant endophytic fungi. Pectinases, cellulases, amylases, laccases, proteases, and lipases are among the enzymes that endophytic fungi create as a defense mechanism against pathogenic organisms and to obtain nutrients from the host.^23^ Due to their ability to produce a wide range of enzymes, these fungi can be used in a variety of industries, including food, cosmetics, cleaning agents, biofuels, and pharmaceuticals. Enzyme metabolites that were extracted from endophytic fungi exhibited a range of pharmacological characteristics, including anticancer, antimicrobial, antioxidant, anti-inflammatory, and antidiabetic effects.^24^

The Tamaricaceae family includes the Nile Tamarisk (*Tamarix nilotica*), commonly known as Turfaa in Arabic. Based on phytochemical analysis, the main chemical components of *Tamarix nilotica* are flavonoids, tannins, and phenolics. Egyptian traditional herbal medicine uses this plant for several uses, including as the treatment of headaches, inflammation, and infections.^25^ Studying the target plant’s endophytes is essential to maximizing the use of the secondary metabolites produced by the plant.^26^ Particularly in semi-arid and desert regions, *Tamarix* species exhibit remarkable adaptability to climate change. Consequently, it is anticipated that these species will host a diverse array of microbial endophytes.

As a result, the major objective of this study was to isolate and identify culturable fungal endophytes in *Tamarix nilotica* from the Taif area (Saudi Arabia). Additionally, the endophytic isolates’ extracellular enzymatic production (amylase, cellulase, protease, and lipase) and antifungal activity were investigated.

## Materials and methods

### Sampling

Root and stem samples (40 samples) from 20 *Tamarix nilotica* plants were obtained at various places in Taif, Saudi Arabia. Healthy, mature plants were carefully selected for sampling. Plant samples were placed in sterile plastic bags for storage at 4°C until use.^27^

### Isolation of endophytic fungi

Forty root and stem samples (20 each) were thoroughly cleaned with running tap water for 10 min before being sliced into 1 cm slices. Four representative portions from each plant sample (for a total of 40 samples) were used. To surface sterilize, the plant pieces were soaked in 70% ethanol for 1 min, then in a 0.5% sodium hypochlorite solution for 3 min and 70% ethanol for 30 s before being washed three times with sterile distilled water.^28^ They were left to dry on paper towels. After drying, the plant pieces (4) were placed on potato dextrose agar (PDA) plates from HiMedia (India) with 50 mg/L chloramphenicol to avoid bacterial growth. All plates were incubated at 25°C for 7–10 d to isolate endophytic fungi. Individual colonies from PDA plates were chosen and transferred to fresh PDA medium before being incubated at 26°C for 10 d to produce pure fungal isolates.^29^ Micro-cultivations were performed to identify endophytic fungi, and macro- and micro-morphological aspects of the somatic and reproductive structures were observed using specific methodology and literature.^30–32^ The colonization frequency (CF) and dominant fungal percentages of endophytic fungi were calculated.^33,34^ CF (%) = (number of segments colonized by endophytes/total number of segments investigated) × 100.

### Molecular identification of fungal isolates

The genomic DNA was isolated using the Gashgari and Gherbawy^15^ approach. The internal transcribed spacer (ITS) region of ribosomal DNA (rDNA) was amplified by PCR using primers ITS1-F (CTTGGTCATTTAGAGGAAGTAA) and ITS4 (TCCTCCGCTTATTGATATGC).^35,36^ In a final volume of 50 μl, PCR amplifications were carried out using 2 μl of DNA with 0.5 μM of each primer, 150 μM of dNTP, 1 U of Taq DNA polymerase (Promega), and reaction buffer. Amplification was performed in a thermal cycler with an initial denaturation of 3 min at 94°C, followed by 35 cycles of 1 min at 94°C, 1 min at 50°C, 1 min at 72°C, and a final extension of 10 min at 72°C. Aliquots of PCR products were electrophoresed on a 1% agarose gel, stained with ethidium bromide, and observed using UV transillumination. ExoSAP-IT (USB Corporation, under license from GE Healthcare) purified the PCR products following one manufacturer’s directions. The purified products were sequenced using an automated DNA sequencer (ABI PRISM 3700) and the BigDye Deoxy Terminator cycle-sequencing kit (Applied Biosystems) according to the manufacturer’s instructions. Sequences were submitted to GenBank through the NCBI website (http://www.ncbi.nlm.nih.gov). The sequences acquired in this investigation were compared to the GenBank database using the BLAST software available on the NCBI website (http://www.ncbi.nlm.nih.gov/BLAST). DNA sequences were aligned first using Clustal X 1.81.^37^ TREECON^38^ for Windows (version 1.3b, 1998) was used to build a neighbor-joining tree using the Jukes-Cantor model,^39^ with 100 bootstrap replications.

### Screening of endophytic fungi for the antifungal activities

Dual-culture technique was used to investigate the antifungal activity of the endophytic fungi against two plant pathogenic fungi.^40^ The assay was performed on potato dextrose agar as it favors the growth of the pathogenic fungi – *Fusarium solani* and *Rhizoctonia solani*. Mycelia agar discs (5 mm diameter) from endophytic and pathogenic fungi cultures were inoculated on PDA plate at the periphery, opposite to each other. Petri plates inoculated only with test pathogens served as control. The experiment was done in triplicates. Paired cultures and control plates incubated at 25°C for 5–7 d were observed, and antagonism was expressed as percentage growth inhibition. The percentage of inhibition was calculated from the following equation: Inhibition (%) = [(growth diameter in the control sample – growth diameter in the sample with treated endophytes) × 100]/growth diameter in the control sample.^41^

### Screening of endophytic fungi for amylase, cellulase, protease, and lipase production on solid media

#### Amylase assay

Endophytic fungal cultures grown on PDA were sliced into 5 mm discs using a borer. Three discs were placed on a Petri plate containing 15 mL of glucose yeast extract peptone agar (GYP) medium (glucose 1 g, yeast extract 0.1 g, peptone 0.5 g, agar 16 g, and 1000 mL of distilled water) with 0.2% soluble starch at pH 6.0.^42^ Following incubation, the plates were flooded with a 1% iodine solution in 2% potassium iodide (Iodine has a low solubility in water, but it dissolves much more quickly in an aqueous solution containing iodide ions because tri-iodide ions are produced. So, the KI simply enhances the solubility of the iodine in the solution). The zone of clearance surrounding the colony was measured.^43^

#### Cellulase assay

For cellulose activity experiments, the plates were flooded with 1 mg/ml Congo red solution, which was drained out after 15 min, followed by 15 min of flooding with 1 M NaCl. The presence of a distinct halo around the colony on a red backdrop indicated positive cellulase activity. Enzyme activities were assessed as the distance in millimeters from the well’s edge to the halo, normalized by the sample’s total protein content.

#### Protease assay

For cellulose activity experiments, the plates were flooded with 1 mg/ml Congo red solution, which was drained out after 15 min, followed by 15 min of flooding with 1 M NaCl. The presence of a distinct halo around the colony on a red backdrop indicated positive cellulase activity. Enzyme activities were assessed as the distance in millimeters from the well’s edge to the halo, normalized by the sample’s total protein content.

#### Lipase assay

For screening, a qualitative plate assay was used, as described before for selecting lipase-producing bacteria.^44^ Isolates plugs were inoculated on tributyrin agar plates and incubated at 30°C for 5 d. Lipase hydrolyzed tributyrin, resulting in a clear zone.

## Results and discussion

### *Endophytic fungi associated with* Tamarix nilotica

This is the first investigation of the fungal endophytes of the *Tamarix nilotica* plant discovered in Taif, Saudi Arabia. A total of 160 plant (root and stem) segments from 20 different plants were examined for endophytic fungi. Mycelium emerged from 49 of 160 segments, accounting for a total colonization rate of 30.6%. Gashgari et al.^45^ researched endophytic fungi associated with medicinal plants found in salt marshes in the Al-Jouf area of Saudi Arabia, including *Tamarix nilotica*. This plant displayed the most variation in endophytic fungi, with a relative frequency of 27.3%. In the Taif area, Gherbawy and Elhariry^46^ discovered that mycelium of endophytic fungus developed from 120 out of 400 twig segments of *Juniperus procera* plant, yielding an overall colonization rate of 30%, whereas *Calotropis procera* had an overall foliar colonization rate of 35.1%.

Based on morphological features, 49 isolates were found and categorized into 21 distinct operational taxonomic units (OTUs) (Table 1). The total number of endophytic fungi isolated from the root and stem segments was 33 and 16 (out of 160), with 17 and 12 fungal species (out of 21 fungal species collected from all plant parts, respectively).

**Table 1.** Isolated and identified endophytes from *Tamarix nilotica* in relationship with the genus or species and the identity percentage found in the NCBI GenBank website.

Max. identity (%)	Closely related fungal sequence	Accession numbers	Isolate code	No
99	*Alternaria alternata* MN044802	PP779480	TUAa1	1
100	*Aspergillus ochraceus* MN088855	PP779481	TUAo1	2
100	*Aspergillus sydowii* KX958061	PP779482	TUAs1	3
99	*Aspergillus terreus* OR52860	PP779483	TuAt1	4
100	*Eupenicillium crustaceum* HF54639	PP779484	TUEc1	5
98.4	*Fungal* sp. OP679904	PP779485	TUFs1	6
99.4	*Fungal* sp. EU781672	PP779486	TUFS2	7
99.4	*Fungal* sp. OP679904	PP779487	TUFs3	8
98.6	*Fungal* sp. OP679904	PP779488	TUFs4	9
98.9	*Fungal* sp. OP679904	PP779489	TUFs5	10
98.9	*Fungal* sp. OP679904	PP779490	TUFs6	11
98.3	*Fungal* sp. OP679904	PP779491	TUFs7	12
99.0	*Fungal* sp. OP679904	PP779492	TUFs8	13
99.1	*Fungal* sp. OP679904	PP779493	TUFs9	14
99.1	*Fungal* sp. EU781672	PP779494	TUFs10	15
98.9	*Fungal* sp. *EU781672*	PP779495	TUFs11	16
98.6	*Fungal* sp. Kx271288	PP779496	TUFs12	17
99.6	*Fusarium brachygibbosum* KJ541486	PP779497	TUFb1	18
99.7	*Fusarium oxysporum* MT453296	PP779498	TUFo1	19
*99.7*	*Penicillim chrysogenum LT558875*	PP779499	TUPc1	20
99.2	*Scopulariopsis* sp. HF546378	PP779500	TUSs1	21

**Figure 1.**
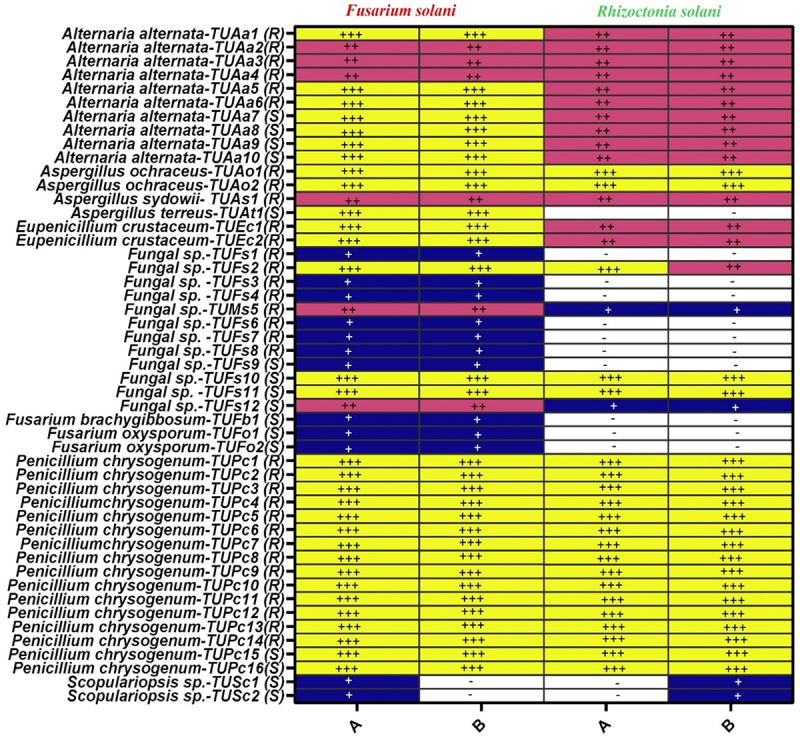
Antifungal activities of normal and boiled filtrate of 49 fungal endophytes isolates from *Tamarix nilotica* against *Fusarium solani* and *Rhizoctonia solani*.

**Figure 2.**
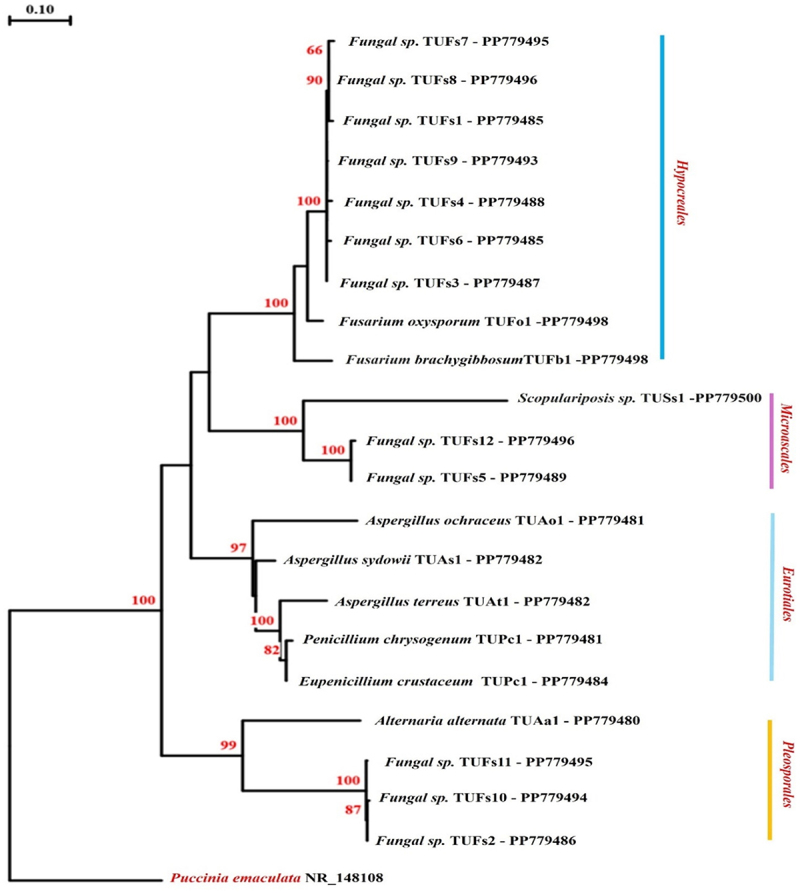
Phylogenetic tree based on the ITS region of rRNA showing closest relatives of fungal endophytes isolated from *Tamarix nilotica*. The tree was constructed by neighbor-joining algorithm using maximum composite likelihood model. Bootstrap percentages from 100 replicates are shown. The tree was rooted with *Puccinia emaculata* [NR_148108] as the out-group.

From 160 plant segments, 49 different fungi endophytes were found. There were 21 distinct OTUs identified by rRNA’s ITS sequence (Table 1). Of the 49 isolates recovered, 12 sterile mycelia isolates were recognized as fungal species (TUFs 1–12). They were divided into three categories depending on molecular techniques. The first clade includes seven fungal species (TUFs 1, 3, 4, 6, 7, 8, and 9) that were clustered with *Fusarium oxysporum* and *F. brachygibbosum* with a 100% bootstrap factor, showing that these species may belong to Hypocreales. The second clade comprises TUFS5 and TUFs 12, which were completely bootstrapped using *Scopulariopsis* sp from the Microascales. Three fungal species (TUFs2, 10, and 11), along with *Alternaria alternata*, formed the third clade, with a 99% bootstrap factor confirming Plerosporales (Figure 1). Based on these findings, not all endophytic fungi can be identified to the species or genus level using GenBank data, as previously reported by Gherbawy and Elhariry.^46^ Furthermore, Amirita et al.^47^ discovered that sterile forms had the largest overall relative proportion of occurrence (48%), followed by Hyphomycetes (25%), Coelomycetes (14%), and Xylariales (13%). According to Lacap et al.,^48^ sterile mycelia are found in the vast majority of endophytic investigations. All of the species discovered in this study are classed as Ascomycota, which contains four orders: Eurotiales, Hypocreales, Microascales, and Pleosporales (Figure 2). These findings were consistent with previous studies,^49,50^ which showed that fungal endophytes are especially common in Ascomycota.

A: Boiled filtrate of endophytic isolates; B: Normal filtrate of endophytic isolates Inhibition zone: + >2 mm; ++, 2–10 mm; +++; <10 mm. No effect, -.

**Figure 3.**
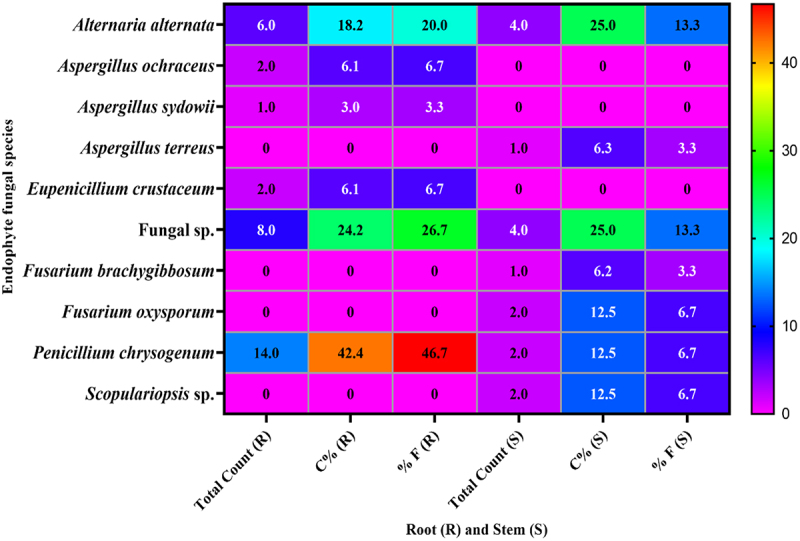
Total count (C), Percentage of C, Frequency of endophytic fungi isolated from 30 samples of root and stem of *Tamarix nilotica* on PDA medium at 26° ± 2°C.

*Penicillium chrysogenum* was the most prevalent of the 17 fungal species found on root segments, accounting for 42.4% of the total count and 46.7% of the frequency. *Alternaria alternata* finished second, accounting for 18.2% of the total counts and 20% of the frequency percentage (Figure 3). The two most frequent species identified in stem samples were *Alternaria alternata* and fungi sp. In terms of frequency percentages and overall count, they each contributed 13.3% and 25%, respectively. According to Sarma and Hyde,^51^ “core-group fungi” are those that have a frequency of ≥1%. These dominant endophytic fungi have the potential to significantly alter plant fitness. *Penicillium chrysogenum* was previously the most often isolated species, colonizing 98.57% of seven medicinal plants, including *Tamarix nilotica*, in Saudi Arabian salt marshes.^45^ Aside from *Penicillium chrysogenum*, the most common species were Mycelia sterilia (92.86%), *Aspergillus ochraceus* (42.86%), and *Alternaria alternata* (32.86%). Recently, 35 endophytic fungal isolates were discovered in Azerbaijan by Ebadi et al.^29^ from root pieces of *Tamarix ramosissima*. Based on their morphological attributes, these isolates were categorized into nine categories. Using DNA sequencing, these isolates are categorized into nine species and four genera: *Aspergillus bicephalus*, *Alternaria alternata, Fumigatus, A. niger, A. Terreus, Fusarium oxysporum, F. solani, F. redolens*, and *Talaromyces allahabadensis*. Microorganisms can penetrate the plant and form endophytes, which can subsequently colonize various belowground and aboveground plant organs as the plant grows.^52,53,^^54^ According to this research, there are more endophytic fungal species (33 isolates) in healthy *Tamarix* roots than in stem segments (16 isolates). Cao et al.^55^ isolated 163 and 68 endophytic fungal strains from 200 to 100 leaves and roots of Musa acuminata plants, respectively. Abdel-Motaal et al.^41^ found 13 and 15 endophytic fungus species from *Hyoscyamus muticus* stem and root, respectively.

### Antifungal activity screening

Overall, 100% and 87.76% of the endophytic fungal isolates showed antifungal efficacy against *Fusarium solani* and *Rhizoctonia solani* (Figure 1). According to Gherbawy and Ghashgari,^56^ 20% of the identified endophytic species from *Calotropis procera* in Saudi Arabia have antifungal activity against at least one of the pathogenic fungi investigated. Comparable percentages of 30% were found in other investigations.^21,57–59^ Except for six isolates from: *Aspergillus terreus* (1 isolate), *Fusarium brachygibbosum* (1), *F. oxysporum* (2), and *Scopulariopsis* sp. (2), which had no inhibitory ability against *R. solani* (Figure 1), all the tested isolates (49) had varying inhibitory effects against pathogenic fungi (*F. solani* and *R. solani*).

Sixteen isolates (out of 49 examined) from *Penicillium chrysogenum* (16 isolates), *Eupenicillium crustaceum* (2), *Aspergillus ochraceus*, *A. terreus* (1), and Fungal sp. 10 & 11 (2) shown 100% antifungal activity independent of the source of isolation, root or stem sample (Figure 1). *Aspergillus sydowii* (1 isolate), *Eupenicillium crustaceum* (2), and *Penicillium chrysogenum* (16) demonstrated the most inhibitory effects on *R. solani*. These findings were congruent with those of Gashgari et al.,^45^ who investigated the antifungal activity of endophytic fungi isolated from medicinal plants found in the Al-Jouf. Their results showed that the isolates *P. chrysogenum, E. crustaceum, F. brachygibbosum, A. sydowii*, and *P. chrysogenum* exhibited strong inhibition against *F. oxysporum, F. solani*, and *Alternaria alternata*, plant pathogenic fungi.

The results of this study revealed that boiling fungal filtrates had no influence on their antifungal capabilities; these findings confirmed the presence of thermostable antifungal chemicals in the screened isolates rather than proteinaceous components.^60^ In addition, the studied isolates showed varied degrees of inhibition against pathogenic fungi (Figure 1). The ability of isolates from the same species to produce metabolites with antimicrobial activity varied greatly, according to Pelaez et al.,^61^ who screened 187 endophytic fungi derived from nine plant species that grew on saline and gypsum soils in central Spain for antimicrobial activity production. Additionally, Park et al.^20^ discovered that while several endophytic fungi exhibited identical colony morphology, their in vivo antifungal effectiveness against six fungal plant pathogens varied. Likewise, these findings indicated that many isolates per species should be examined while studying biological activities (Figure 3).

### Hydrolytic enzyme activities

**Figure 4.**
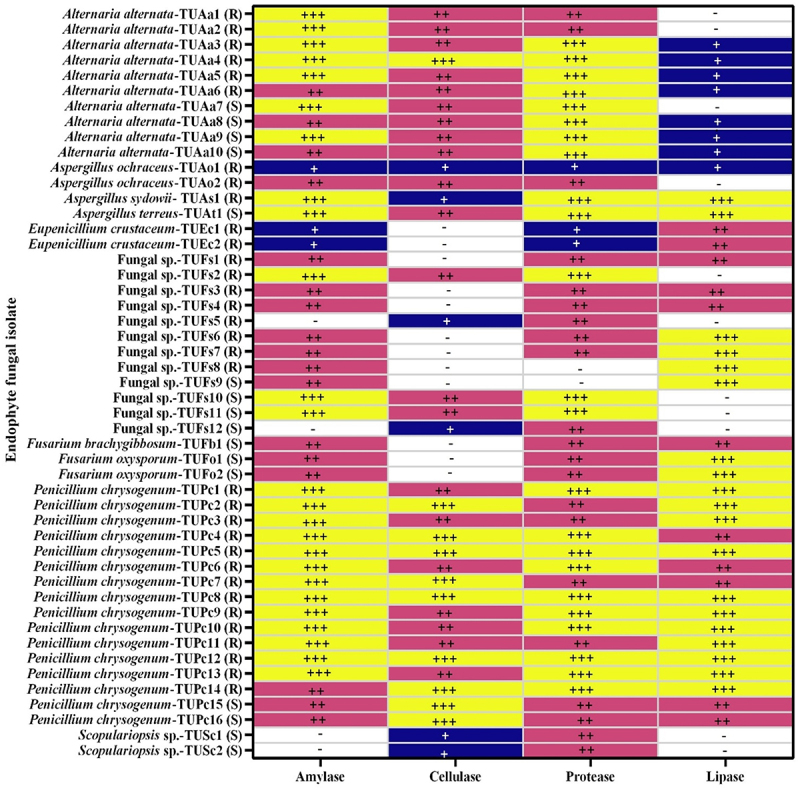
Evaluation of extracellular hydrolytic enzyme activities from 49 fungal endophytes isolates isolated from *Tamarix nilotica.*

In many different industries, such as paper and pulp, food and beverage, detergents, medicines, and leather processing, enzymes exhibit great potential. Enzymes have been extracted from a variety of sources, including plants and animals, for many decades. Microorganisms are becoming a more viable source for commercial enzyme synthesis due to their ubiquitous availability and quick growth rate.^62^ Endophytic fungi in plant tissues produce a wide range of secondary metabolites and enzymes that serve numerous biological functions. All 49 endophytic fungal isolates obtained in this study were examined for their capacity to produce extracellular enzymes (amylase, cellulase, protease, and lipase), as seen in Figure 1. All of the isolates tested showed variable levels of enzymatic activity. Twenty-six isolates showed enzymatic activity for all the enzymes tested, and 17 isolates were able to produce three of the enzymes. Only six of the isolates could produce one of the enzymes tested (Figure 4).

-, implies no halo zone indicates no enzyme activity; +, implies 2 mm diameter of the halo zone indicates enzyme activity; ++, implies 4 mm halo zone indicates enzyme activity; +++, implies 6 mm diameter of the halo zone indicates enzyme activity.

*Alternaria alternata* (7 isolates), *Aspergillus sydowii* (1), *A. terreus* (1), Fungal sp. 1 (1), Fungal sp. 10 (1), Fungal sp. 11 (1), and *Penicillium chrysogenum* (13 isolates) were the most amylase producers. The percentage of cellulase-producing isolates was 75.5%, with the most cellulolytic isolates coming from *Alternaria alternata* (1 out of 10) and *Penicillium chrysogenum* (9 out of 16). Endophytic isolates from many indigenous medicinal plants in India generated 62.5% amylase, 12.5% cellulase, and 29.2% lipase.^63–65^ It has previously been reported that *Alternaria alternata, Aspergillus sydowii*, and *Penicillium chrysogenum* are cellulase and amylase producers.^66–70^

Figure 4 reveals that the endophytic organisms used in this study exhibit 98.9% protease activity and 77.6% lipase activity. *Alternaria alternata* (8), *Aspergillus sydowii* (1), *Aspergillus terreus* (1), and *Penicillium chrysogenum* (10) were the most protease-producing isolates, while *Aspergillus sydowii* (1), *Aspergillus terreus* (1), Fungal sp. 6–9, *Fusarium oxysporum* (2), and *Penicillium chrysogenum* (11) were the most lipolytic producers. The proteolytic and lipolytic activities of the above-mentioned fungal species were tested using several searches.^71,72,^^73–78,79^

In conclusion, 21 different OTUs of endophytic fungi were found in *Tamarix* plants, with the most frequently isolated species being *Alternaria alternata* (10 isolates), *Penicillium chrysogenum* (16 isolates), and Fungal sp. (12). Furthermore, 42.9% of the endophytic fungi isolated from *Tamarix nilotica* have antifungal activity against *Fusarium solani* and *Rhizoctonia solani*. Furthermore, the endophytic species isolated from *Tamarix nilotica* exhibited varied levels of extracellular hydrolytic enzyme production. Out of 49 isolates, 21 demonstrated the highest levels of amylase, cellulase, protease, and lipase activity. The study’s findings revealed that endophytic fungus isolated from *Tamarix nilotica* plants may be used to produce bioactive components such as enzymes and antifungal chemicals, potentially leading to advancements in eco-friendly biotechnology.

## Data Availability

All data generated or analyzed during this study were included in this manuscript.

## References

[1] Azevedo J, Walter M, Pereira J, Welington A. Endophytic microorganisms: a review on insect control and recent advances on tropical plants. Electron J Biotechnol. 2000;3(1):41–9. doi:10.2225/vol3-issue1-fulltext-4.

[2] Potshangbam M, Devi SI, Sahoo D, Strobel GA. Functional characterization of endophytic fungal community associated with Oryza sativa L. and Zea mays L. Front Microbiol. 2017; Sec. Fungi and Their Interactions. Volume 8. doi:10.3389/fmicb.2017.00325. PMC5332368 28303127

[3] Rodriguez RJ, White JF, Arnold AE, Redman RS. Fungal endophytes: diversity and functional roles. New Phytol. 2009;182(2):314–330. doi:10.1111/j.1469-8137.2009.02773.x. 19236579

[4] Aguilar-Trigueros CA, Rillig MC. Effect of different root endophytic fungi on plant community structure in experimental microcosms. Ecol Evol. 2016;6(22):8149–8158. doi:10.1002/ece3.2416. 27878084 PMC5108266

[5] Backman PA, Sikora RA. Endophytes: an emerging tool for biological control. Biol Control. 2008;46(1):1–3. doi:10.1016/j.biocontrol.2008.03.009.

[6] Hassani MA, Durán P, Hacquard S. Microbial interactions within the plant holobiont. Microbiome. 2018;6(1):58–74. doi:10.1186/s40168-018-0445-0. 29587885 PMC5870681

[7] Mwanga Z, Mvungi E, Tibuhwa D. Antimicrobial activities of endophytic fungi secondary metabolites from moringa oleifera (lam). Tanzania J Sci. 2019;45:463–476.

[8] Selim KA, El-Beih AA, Abdel-Rahman TM, El-Diwany A. Biodiversity and antimicrobial activity of endophytes associated with Egyptian medicinal plants. Mycoloshe. 2011;2(6):669–678. doi:10.5943/mycosphere/2/6/7.

[9] Cheng C, Li D, Qi Q, Sun X, Anue MR, David BM, Zhang Y, Hao X, Zhang Z, Lai Z. The root endophytic fungus serendipita indica improves resistance of banana to Fusarium oxysporum f. sp. cubense tropical race 4. Eur J Plant Pathol. 2020;156(1):87–100. doi:10.1007/s10658-019-01863-3.

[10] Fontana DC, de Paula S, Torres AG, de Souza VHM, Pascholati SF, Schmidt D, Dourado Neto D. Endophytic fungi: biological control and induced resistance to phytopathogens and abiotic stresses. Pathogens. 2021;10(5):570–598. doi:10.3390/pathogens10050570. 34066672 PMC8151296

[11] Latz MAC, Jensen B, Collinge DB, Jørgensen HJL. Endophytic fungi as biocontrol agents: elucidating mechanisms in disease suppression. Plant Ecol Divers. 2018;11(5–6):555–567. doi:10.1080/17550874.2018.1534146.

[12] Guo B, Wang Y, Sun X, Tang K. Bioactive natural products from endophytes: a review. Appl Biochem Microbiol. 2008;44(2):136–142. doi:10.1134/S0003683808020026. 18669256

[13] Dos Santos IP, da Silva LC, da Silva MV, de Araújo JM, Cavalcanti Mda S, Lima VL. Antibacterial activity of endophytic fungi from leaves of Indigofera suffruticosa Miller (Fabaceae). Front Microbiol. 2015;6(6):350–356. doi:10.3389/fmicb.2015.00350. 25999918 PMC4423342

[14] Gangadevi V, Sethumeenal S, Yogeswari S, Rani G. Screening endophytic fungi isolated from a medicinal plant, Acalypha indica L. for antibacterial activity. Ind J Sci Technol. 2008;1(5):1–6. doi:10.17485/ijst/2008/v1i5.11.

[15] Gherbawy YA, Gashgari RM. Molecular characterization of fungal endophytes from Calotropis procera plants in Taif region (Saudi Arabia) and their antifungal activities. Plant Biosys. 2014;148(6):1085–1092. doi:10.1080/11263504.2013.819043.

[16] Idris A, Al-Tahir I, Idris E. Antibacterial activity of endophytic fungi extracts from the medicinal plant Kigelia Africana. Egypt Acad J Biol Sci. 2013;5(1):1–9. doi:10.21608/eajbsg.2013.16639.

[17] Liang H, Xing Y, Chen J, Zhang D, Guo S, Wang C. Antimicrobial activities of endophytic fungi isolated from Ophiopogon japonicus (Liliaceae). BMC Complement Altern Med. 2012;12(1):238. doi:10.1186/1472-6882-12-238. 23190550 PMC3534486

[18] Mamarasulov B, Davranov K, Umruzaqov A, Ercisli S, Alharbi SA, Ansari MJ, Krivosudská E, Datta R, Jabborova D. Evaluation of the antimicrobial and antifungal activity of endophytic bacterial crude extracts from medicinal plant Ajuga turkestanica (Rgl.) brig (Lamiaceae). J King Saud Univ Sci. 2023;35(4):102644–103647. doi:10.1016/j.jksus.2023.102644.

[19] Mapperson RR, Kotiw M, Davis RA, Dearnaley JDW. The diversity and antimicrobial activity of Preussia sp. endophytes isolated from Australian dry rainforests. Curr Microbiol. 2014;68(1):30–37 doi:10.1007/s00284-013-0415-5. 23975673

[20] Park J-H, Park JH, Choi GJ, Lee SW, Jang KS, Choi YH, Cho KY, Kim J-C. Screening for antifungal endophytic fungi against six plant pathogenic fungi. Mycobiol. 2003;31(3):179–182. doi:10.4489/MYCO.2003.31.3.179.

[21] Phongpaichit S, Rungjindama N, Rukachaisirikul V, Sakayaroj J. Antimicrobial activity in cultures of endophytic fungi isolated from Garcinia species. FEMS Immunol Med Microbiol. 2006;48(3):367–372. doi:10.1111/j.1574-695X.2006.00155.x. 17052267

[22] Raviraja NS, Maria GL, Sridhar KR. Antimicrobial evaluation of endophytic fungi inhabiting medicinal plants of the Western Ghats of India. Eng Life Sci. 2006;6(5):515–520. doi:10.1002/elsc.200620145.

[23] Vasundhara M, Reddy MS, Kumar A. Secondary metabolites from endophytic fungi and their biological activities. In: Gupta V Pandey A. editors. New and future developments in microbial biotechnology and bioengineering. Elsevier; 2019. p. 237–258. doi:10.1016/b978-0-444-63504-4.00018-9.

[24] Darwish AMG, Balbool M, Abo Nouh FA. Industrially important enzymes of endophytic fungi. In: Endophytic Fungi The Full Story of the Untapped Treasure Developments in Applied Microbiology and Biotechnology. Academic Press; 2024. p. 157–179. doi:10.1016/B978-0-323-99314-2.00014-0.

[25] Abdelgawad AAM. Tamarix nilotica (Ehrenb) bunge: a review of phytochemistry and pharmacology. J Microb Biochem Tech. 2017;9:544–553.

[26] Jha P, Kaur T, Chhabra I, Panja A, Paul S, Kumar V, Malik T. Endophytic fungi: hidden treasure chest of antimicrobial metabolites interrelationship of endophytes and metabolites. Front. Microbiol. 2023;14:1227830. doi:10.3389/fmicb.2023.1227830. 37497538 PMC10366620

[27] Lu Y, Chen C, Chen H, Zhang J, Chen W. Isolation and identiﬁcation of endophytic fungi from actinidia macrosperma and investigation of their bioactivities. Evidence-Based Complementary & Alternative Med. 2012;2012:382742. doi:10.1155/2012/382742. PMC3235672 22203869

[28] Arnold AE, Maynard Z, Gilbert GS, Coley PD, Kursar TA. Are tropical fungal endophytes hyper diverse? Eco Let. 2000;3(4):267–274. doi:10.1046/j.1461-0248.2000.00159.x.

[29] Ebadi M, Najari S, Miandoab LZ, Chaparzadeh N, Ebadi A. Mining Tamarix ramosissima roots for endophytic growth promoting fungi to improve wheat root growth. Res Sq [Prepr]. 2024;29:rs.3.rs–4277791. doi:10.21203/rs.3.rs-4277791/v1. PMID: 38746082; PMCID: PMC11092856.

[30] Domsch KH, Gams W, Anderson T-H. Compendium of soil fungi. 2nd ed. London: Academic Press; 2007.

[31] Leslie J, Summerell BA. The fusarium laboratory manual. (IA): Blackwell Publishing; 2006.

[32] Samson RA, Frisvad JC. Penicillium subgenus Penicillium: new taxonomic schemes, mycotoxins and other extrolites. Stud In Mycol. 2004;49:1–260.

[33] Kumar D, Hyde KD. Biodiversity and tissue-recurrence of endophytic fungi in Tripterygium wilfordii. Fungal Divers. 2004;2006(17):69–90.

[34] Petrini O, Fisher PJA. Comparative study of fungal endophytes in xylem and whole stem of Pinus sylvestris and Fagus sylvatica. Trans Br Mycol Soc. 1988;91(2):233–238. doi:10.1016/S0007-1536(88)80210-9.

[35] Gardes M, Bruns TD. ITS primers with enhanced specificity for basidiomycetes – application to the identification of mycorrhizae and rusts. Mol Ecol. 1993;2(2):113–118. doi:10.1111/j.1365-294X.1993.tb00005.x. 8180733

[36] White TJ, Bruns T, Lee S, Taylor J. Amplification and direct sequencing of fungal ribosomal RNA genes for phylogenetics. In: Innis M, Gelfand D, Sninsky J White T. editors. PCR protocols: a guide to methods and applications. San Diego (CA): Academic Press; 1990. p. 315–322.

[37] Thompson JD, Gibson TJ, Plewniak F, Jeanmougin F, Higgins DG. The clustal X windows interface: flexible strategies for multiple sequence alignment aided by quality analysis tools. Nucleic Acids Res. 1997;25(24):4876–4882. doi:10.1093/nar/25.24.4876. 9396791 PMC147148

[38] Van de Peer Y, De Wachter R. TREECON for windows: a software package for the construction and drawing of evolutionary trees for the Microsoft windows environment. Bioinformatics. 1994;10(5):569–570. doi:10.1093/bioinformatics/10.5.569. 7828077

[39] Jukes TH, Cantor CR. Evolution of protein molecules. In: Munro H. editor. Mammalian protein metabolism. Vol. 3. (NY): Academic Press; 1969. p. 21–32.

[40] Oldenburg KR, Vo KT, Ruhland B, Schatz PJ, Yuan Z. A dual culture assay for detection of antimicrobial activity. J Biomol Screen. 1996;1(3):123–130. doi:10.1177/108705719600100305.

[41] Abdel-Motaal FF, Nassar MSM, El-Zayat SA, El-Sayed MA, Ito S-I. Antifungal activity of endophytic fungi isolated from Egyptian henbane (Hyoscyamus muticusL.). J Bot. 2010;42(4):2883–2894.

[42] Kathiresan K, Manivannan S. α-amylase production by penicillium fellutanum isolated from mangrove rhizosphere soil. Afr J Biotechnol. 2006;5:829–832.

[43] Maria GL, Sridhar KR, Raviraj NS. Antimicrobial and enzyme activity of mangrove endophytic fungi of southwest coast of India. J Agric Technol. 2005;1:67–80.

[44] Patel BG, Shah RK. Biodegradation of cotton seed soap stocks by novel indigenous bacillus species. Biosci Biotech Res Comm. 2018;11(3):505–511. doi:10.21786/bbrc/11.3/21.

[45] Gashgari R, Gherbawy Y, Ameen F, Alsharari S. Molecular characterization and analysis of antimicrobial activity of endophytic fungi from medicinal plants in Saudi Arabia. Jundishapur J Microbiol. 2016;9(1):e26157. doi:10.5812/jjm.26157. PMID: 27099679; PMCID: PMC4834135. 27099679 PMC4834135

[46] Gherbawy YA, Elhariry HM. Endophytic fungi associated with high-altitude Juniperus trees and their antimicrobial activities. Plant Biosys. 2016;150(1):131–140. doi:10.1080/11263504.2014.984011.

[47] Amirita SP, Swetha J, Vasanthi NS, Kannan KP. Enumeration of endophytic fungi from medicinal plants and screening of extracellular enzymes. World J Sci Tech. 2013;2:13–19.

[48] Lacap DC, Hyde KD, Liew ECY. An evaluation of the fungal ‘morphotype’ concept based on ribosomal DNA sequence’. Fungal Divers. 2003;12:53–66.

[49] Gehlot P, Bohra NK, Purohit DK. Endophytic mycoflora of inner bark of Prosopis cineraria – a key stone tree species of Indian desert. Am Eur J Bot. 2008;1:01–04.

[50] Pepeljnjak S, Kosalec V, Kaloera Z, Blazevic N. Antimicrobial activity of juniper berry essential oil (Juniperus communis L. Cupressaceae). Acta Pharm. 2005;55(4):417–422. 16375831

[51] Sarma VV, Hyde KD. A review on frequently occurring fungi in mangroves. Fungal Divers. 2001;8:1–34.

[52] Chi F, Shen SH, Cheng HP, Jing YX, Yanni YG, Dazzo FB. Ascending migration of endophytic rhizobia, from roots to leaves, inside rice plants and assessment of benefits to rice growth physiology. Appl Environ Microbiol. 2005;71(11):7271–7278. doi:10.1128/AEM.71.11.7271-7278.2005. 16269768 PMC1287620

[53] Compant S, Kaplan H, Sessitsch A, Nowak J, Barka EA, Clement C. Endophytic colonization of Vitis vinifera L. by Burkholderia phytofirmans strain PsJN: from the rhizosphere to inflorescence tissues. FEMS Microbiol Ecol. 2008;63(1):84–93. doi:10.1111/j.1574-6941.2007.00410.x. 18081592

[54] Wang S, Ma T, Yao X, Yao Z, Wang Z, Dong Z, Luo M, Huang L. Structure of endophytes in the root, stem, and leaf tissues of sweetpotato and their response to sweetpotato scab disease caused by Elsinoë batatas. Agronomy. 2023;13:2965. doi:10.3390/agronomy13122965.

[55] Cao LX, You JL, Zhou SN. Endophytic fungi from Musa acuminata leaves and roots in South China. World J Microbiol Biot. 2002;18(2):169–171. doi:10.1023/A:1014491528811.

[56] Gherbawy YA, Gashgari RM. Molecular characterization of fungal endophytes from Calotropis procera plants in Taif region (Saudi Arabia) and their antifungal activities. Plant Biosyst - an Int J Dealing with All Aspects of Plant Biol. 2013;148(6):1085–1092. doi:10.1080/11263504.2013.819043.

[57] Huang YJ, Wang JF, Li GL, Zheng ZH, Su WJ. Antitumor and antifungal activities in endophytic fungi isolated from pharmaceutical plants Taxus mairei, cephalotaxus fortunei and Torreya grandis. FEMS Immunol Med Microbiol. 2001;31(2):163–167. doi:10.1111/j.1574-695X.2001.tb00513.x. 11549424

[58] Li H, Qing C, Zhang Y, Zhao Z. Screening for endophytic fungi with antitumour and antifungal activities from Chinese medicinal plants. World J Microbiol Biotechnol. 2005;21(8–9):1515–1519. doi:10.1007/s11274-005-7381-4.

[59] Paul NC, Kim WK, Woo SK, Park MS, Yu SH. Fungal endophytes in roots of aralia species and their antifungal activity. Plant Pathol J. 2007;23(4):287–294. doi:10.5423/PPJ.2007.23.4.287.

[60] Xu K, Li XQ, Zhao DL, Zhang P. Antifungal secondary metabolites produced by the fungal endophytes: chemical diversity and potential use in the development of biopesticides. Front Microbiol. 2012;12:689527. doi:10.3389/fmicb.2021.689527. PMC8255633 34234763

[61] Pelaez F, Collado J, Arenal F, Basilio A, Cabello MA, Dıez MT, García JB, Del Val AG, González V, Gorrochategui J, et al. Endophytic fungi from plants living on gypsum soils as a source of secondary metabolites with antimicrobial. activity. Mycol Res. 1998;102(6):755–761. doi:10.1017/S0953756297005662.

[62] Shankar S, Shikha Gupta M. Microbial xylanases: production, applications, and challenges. In: Gupta V, Sharma G, Tuohy M Gaur R. editors. The handbook of microbial bioresources. CABI Digital Library; 2016. p. 313–330. doi:10.1079/9781780645216.0313.

[63] Jalgaonwala RE, Mahajan RT. Evaluation of hydrolytic enzyme activities of endophytes from some indigenous medicinal plants. J Agri Tech. 2011;7(6):1733–1741.

[64] Nayab DE, Akhtar S, Bangash N, Nisa WU, Hayat MT, Zulfiqar A, Niaz M, Qayyum A, Syed A, Bahkali AH, et al. Production of glucoamylase from novel strain of Alternaria alternata under solid state fermentation. Biomed Res Int. 2022;27:1–9. doi:10.1155/2022/2943790. PMC9633194 36337838

[65] Unal A. Production of -amylase from some thermophilic aspergillus species and optimization of its culture medium and enzyme activity. Afr J Biotechnol. 2015;14(47):3179–3183. doi:10.5897/AJB2015.14924.

[66] Adegoke SA, Odibo FJC. Production, purification and characterization of α-amylase of Aspergillus sydowii IMI 502692. Plant Cell Biotech Mol Biol. 2019;20(23–24):1050–1058.

[67] Dar GH, Kamili AN, Nazir R, Bandh SA, Jan TR, Chishti MZ. Enhanced production of α-amylase by Penicillium chrysogenum in liquid culture by modifying the process parameters. Microb Pathog. 2015;88:10–15. doi:10.1016/j.micpath.2015.07.016. 26220910

[68] Macris BJ. Production and characterization of cellulase and β-Glucosidase from a mutant of Alternaria alternata. Appl Environ Microbiol. 1984;47(3):560–565. doi:10.1128/aem.47.3.560-565.1984. 16346494 PMC239720

[69] Shafique S, Bajwa R, Shafique S. Alpha-amylase production by toxigenic fungi. Nat Prod Res. 2010;24(15):1449–1456. doi:10.1080/14786410903132423. 20812132

[70] Zhang H, Sang Q. Statistical optimization of cellulases production by penicillium chrysogenum QML-2 under solid-state fermentation and primary application to chitosan hydrolysis. World J Microbiol Biotechnol. 2012;28(3):1163–1174. doi:10.1007/s11274-011-0919-8. 22805837

[71] Elwan SH, Ammar MS, Mohawed SM. Lipases from aspergillus sydowi. Zentralbl für Mikrobiol. 1986;141(3):233–239. doi:10.1016/S0232-4393(86)80063-4.

[72] Yadav RP, Saxena RK, Gupta R, Davidson S. Lipase production by Aspergillus and penicillium species. Folia Microbiol (Praha). 1998;43(4):373–378. doi:10.1007/BF02818576. 9821291

[73] Afifi AF, Abo-Elmagd HI, Housseiny MM. Improvement of alkaline protease production by penicillium chrysogenum NRRL 792 through physical and chemical mutation, optimization, characterization and genetic variation between mutant and wild-type strains. Ann Microbiol. 2014;64(2):521–530. doi:10.1007/s13213-013-0685-y.

[74] Osman MS, Khattab OH, Elsaba YM. Aspergillus terreus proteases: characterization and applications. J Chem Biol Phys Sci. 2014;4(3):2333–2346.

[75] Pekkarinen A, Mannonen L, Jones BL, Niku-Paavola M-L. Production of proteases by fusarium species grown on barley grains and in media containing cereal proteins. J Cer Sci. 2000;31(3):253–261.

[76] Sadaf A, Grewal J, Jain I, Kumari A, Khare SK. Stability and structure of penicillium chrysogenum lipase in the presence of organic solvents. Prep Biochem Biotech. 2018;48(10):977–982. doi:10.1080/10826068.2018.1525566. 30461349

[77] Sharma AK, Sharma V, Saxena J. Production of protease and growth characteristics of aspergillus sydowii. Nat & Sci. 2011;9(5):217–221.

[78] Zaferanloo B, Quang TD, Daumoo S, Ghorbani MM, Mahon PJ, Palombo EA. Optimization of protease production by endophytic fungus, Alternaria alternata, isolated from an Australian native plant. World J Microbiol Biotechnol. 2014;30(6):1755–1762. doi:10.1007/s11274-014-1598-z. 24419660

[79] Lu Y, Chen C, Chen H, Zhang J, Chen W. Isolation and identification of endophytic fungi from Actinidia macrosperma and investigation of their bioactivities. Evidence-Based Complement Alternative Med. 2012;2012:382742. doi:10.1155/2012/382742. Epub 2011 Nov 24. PMID: 22203869; PMCID: PMC3235672. PMC3235672 22203869

